# The United States Dissector, or Lessons in Practical Anatomy

**Published:** 1846-11

**Authors:** 


Art. X.—*The United States Dissector, or Lessons in Practical Anatomy.* By Wm. E. Horner, M.D., Professor of Anatomy in the University of Pennsylvania. Fourth edition, with numerous illustrations. Edited by Henry H. Smith, M.D., Fellow of the College of Physicians of Philadelphia, &c. Philadelphia: Lea & Blanchard. 12mo. pp. 444.

Some such guide-book as the above is indispensable to the student in the dissecting room, and this, prepared by one of the most accurate of our anatomists, may claim to combine as many advantages as any other extant. It has been so favorably received that the publishers have issued the fourth edition, which comes forth embellished by various wood cuts. The copy for which we are indebted to the publishers, although received by us a fortnight since, gives proof in its appearance that it has already seen service at the dissecting table, where students have found it a valuable guide.

